# Uterine adenosarcoma associated to lymphovascular emboli: a case report

**DOI:** 10.1186/1757-1626-2-7515

**Published:** 2009-05-18

**Authors:** Rita Sakr, Paul Marzouk, Alexandre Bricou, Fabien Demaria, Annie Cortez, Jean-Louis Benifla

**Affiliations:** 1 Department of Gynecology, Hôpital Armand-Trousseau75012 ParisFrance; 2 Department of Pathology, Hôpital Tenon75020 ParisFrance

## Abstract

**Introduction:**

Uterine adenosarcoma is a rarely observed polypoid tumor with a mixed benign epithelial element and malignant stromal component. The treatment is total hysterectomy with bilateral salpingo-oophorectomy. It could be difficult to diagnose and associated to lymphovascular invasion.

**Case presentation:**

A 45-year-old caucasian uniparous woman presented with uterine bleeding. She had several surgical procedures and pathology of removed recurrent polyps showed no malignancy. Finally, a total abdominal hysterectomy was performed because of atypical cells and suspected uterine adenosarcoma. The hysterectomy specimen confirmed the presence of uterine adenosarcoma associated with lymphatic and vascular tumor emboli. Surgery was completed with a second bilateral salpingo-oophorectomy and pelvic lymphadenectomy.

**Conclusion:**

In our report, we present a case of uterine adenosarcoma which was diagnosed after multiple surgical procedures and associated to lymphovascular emboli known to have a significant impact on overall survival and distant metastasis-free survival.

## Introduction

Uterine adenosarcoma is a mixed epithelial and mesenchymal tumor of the uterus with a benign epithelial element and a malignant stromal component. The lesion is polypoid and sometimes forms multiple polyps. Eventhough it is rarely seen in endometrial biopsy specimens, uterine adenosarcoma is a common neoplasm. The management comprising hysterectomy is curative in most cases but additional bilateral salpingo-oophorectomy is indicated because of the risk of recurrence. The aim of our experience is to show the difficulty of the diagnosis with endometrial biopsies as well as to keep in mind the possible lymphovascular invasion and the additional pelvic lymphadenectomy.

## Case presentation

A 45-year-old Caucasian uniparous woman was referred to our department of gynecology following a suspected diagnosis of adenosarcoma that had been taken from a hysteroscopic resection of an endometrial polyp. She had presented with irregular uterine bleeding of approximately 10 months in duration without dysmenorrhea nor any other symptoms. The gynaecologic examination revealed that the uterus and ovaries were of normal size and that the parametrial tissues were free. The ultrasonographic evaluation showed a small non specific endometrial hyperechogenic 1 cm mass. The pathologic diagnosis of the endometrial tumor removed by surgical hysteroscopy was a probable fibroid polyp. A diagnostic hysteroscopy was performed after 3 months because of uncertain diagnosis and showed a recurrent uterine polyp. Pathologic examination of the endometrium was normal. Magnetic resonance imaging of the pelvis showed a small non-specific endometrial irregularity. The polyp was removed by surgical hysteroscopy and pathologic examination was benign despite few probable atypical stromal cells. According to our surveillance protocol, the performed diagnostic hysteroscopy was normal and the endometrial biopsy showed no malignancy. A new surgical hysteroscopy was performed 2 months later because of irregular uterine bleeding and showed a recurrent uterine polyp. The presence of atypical stromal cells was confirmed by standard examination with Hematoxylin & Eosin Safran of the removed uterine polyp showing enlarged pleiomorphic nuclei. Finally, a total abdominal hysterectomy was performed because of atypical cells and suspected uterine adenosarcoma. The hysterectomy specimen confirmed the presence of uterine adenosarcoma associated to myometrial invasion to the inner half of the myometrium as well as lymphatic and multiple vascular tumor emboli. The histochemical staining for estrogen receptor was negative. Pathologic results of second bilateral salpingo-oophorectomy and pelvic lymphadenectomy were normal. The patient recovered well with no evidence of recurrent disease 12 months after surgery.

## Discussion

Adenosarcoma is a rarely observed mullerian tumor of the uterus and represents 8% of uterine sarcomatous tumors [1]. It was first described in 1974 [2]. It has been mostly reported in post-menopausal women but few cases were observed in women between the ages of 19 and 40 years [3-5]. Clinical symptoms vary from vaginal bleeding to pelvic pain and to protrusion of the tumor from the cervix or vagina [2,6]. The standard management is total hysterectomy and bilateral salpingo-oophorectomy. Moreover, adjuvant chemotherapy and/or radiotherapy were studied in advanced stages because of described cases of metastatic and recurrent uterine adenosarcoma [7]. In most cases, adenosarcoma presents as a yellow to grey endometrial polyp [2]. Morphologically, it is composed of papillary projections. The epithelial element lining the papillary projections is benign, simple cuboidal or columnar, mucinous, ciliated or squamous and the stromal component is malignant [8]. Adenosarcoma may present as recurrent endometrial polyp but the correct diagnosis is only made after pathologic examination identifying the benign epithelial component and the malignant stromal one. Histologic findings were easily confirmed in only 25% of cases [2]. In other cases, it was difficult to differentiate from papillary adenofibromas, mulleroblastomas and carcinosarcomas [2]. Difficulty of diagnosis could be dependent on what areas have been sampled on biopsy and on the fact that it is a rare observed pathology. Increased cellularity of stroma around the epithelial elements forming a cambium layer could be a useful clue suggesting in diagnosis of adenosarcoma [8]. The diagnosis could be sometimes only made or confirmed after multiple pathologic examinations of recurrent polyps. In some cases, many endometrial samples are needed in order to find uterine adenosarcoma [8]. In fact, in our case, the diagnosis of uterine adenosarcoma was difficult and only suspected after multiple histologic analysis of recurrent uterine polyp. Because of suspected lesion, hysterectomy was performed and permitted to confirm histologically the diagnosis of uterine adenosarcoma. In accordance with the routine management of uterine adenosarcoma, bilateral salpingo-oophorectomy was indicated to complete the surgical treatment [2]. Cases of local recurrences were frequent in the literature (50%) and related to bad prognosis most of the times, eventhough the low malignancy component [9]. In our case, lymphovascular emboli were found in the endometrial and myometrial space around the polyp. Bilateral salpingo-oophorectomy and pelvic lymphadenectomy showed no additional lesion in our case. But, concerning vascular and lymphatic space invasion, Rovirosa et al found in a study that it had a significant impact on overall survival and distant disease free survival in early stages [10]. Our study confirms once again that lymphovasular invasion could be associated to uterine adenosarcoma and additional pelvic lymphadenectomy is to be considered in order to complete the surgical treatment.

## Conclusion

Eventhough rare, uterine adenosarcoma is associated to a great risk of local recurrences. Pathologic diagnosis could be difficult and it should be highly suspected in case of recurrent uterine polyps. The management strategy requires confirmed diagnosis and surgery with hysterectomy and bilateral salpingo-oophorectomy in order to avoid any treatment delay and recurrence risk. When associated lymphovascular emboli are described, it seems reasonable to consider pelvic lymphadenectomy since lymphovascular invasion could have impact on overall survival and distant metastasis-free survival.
